# Kyste hydatique de la surrénale: à propos d'un cas

**DOI:** 10.11604/pamj.2015.21.272.6916

**Published:** 2015-08-11

**Authors:** Ammar Mahmoudi, Mezri Maâtouk, Faouzi Noomen, Mohamed Nasr, Khadija Zouari, Abdelaziz Hamdi

**Affiliations:** 1 Service de Chirurgie Générale et Digestive,CHU Fattouma Bourguiba de Monastir, Tunisie; 2 Service d'Imagerie Médicale, CHU Fattouma Bourguiba de Monastir, Tunisie

**Keywords:** kyste hydatique, surrénale, tomodensitométrie, chirurgie, Hydatid cyst, adrenal, CT scan, surgery

## Abstract

Le kyste hydatique de la surrénale reste une affection exceptionnelle et une localisation inhabituelle du kyste hydatique, même dans les pays où l'hydatidose sévit à l’état endémique. Nous rapportons un cas de kyste hydatique surrénalien révélé par des douleurs de l'hypochondre droit. Le diagnostic a été évoqué en préopératoire sur les données de la tomodensitométrie abdominale qui avait objectivé une masse kystique surrénalienne droite. La sérologie hydatique était positive. Le traitement chirurgical avait consisté en une résection du dôme saillant et avait permis de conserver la glande. Le kyste était univésiculaire contenant un liquide eau de roche avec une membrane proligère. Les suites opératoires étaient simples. La surveillance à distance, échographique et immunologique, n'avait pas décelé de récidive avec un recul de deux ans

## Introduction

Le kyste hydatique demeure encore un véritable problème de santé publique dans les pays endémiques. Les localisations hépatiques et pulmonaires sont les plus fréquentes. La localisation surrénalienne reste exceptionnelle et son diagnostic préopératoire peut souvent s'avérer difficile malgré les techniques d'imagerie moderne. Nous rapportons un cas de kyste hydatique surrénalien diagnostiqué avant l'intervention chirurgicale, dont le traitement a permis de conserver la glande et nous essayons de rappeler les caractéristiques cliniques et de préciser l'apport diagnostique de l'imagerie ainsi que les modalités thérapeutiques de cette entité.

## Patient et observation

**Figure 1:**
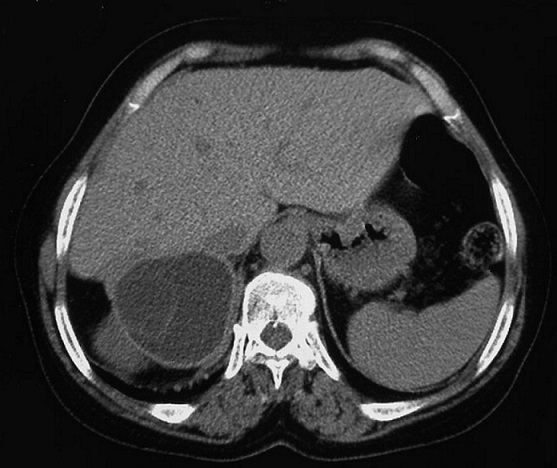
Scanner abdominal en coupe axiale sans injection de produit de contraste: une masse kystique de densité liquidienne homogène présentant une paroi propre de la loge surrénalienne droite

**Figure 2:**
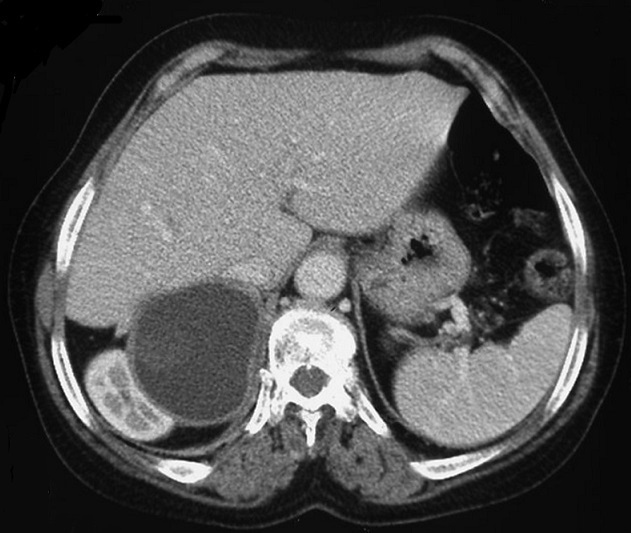
Scanner abdominal en coupe axiale après injection de produit de contraste: Une masse kystique de densité liquidienne homogène présentant une paroi propre non rehaussée après injection de produit de contraste de la loge surrénalienne droite refoulant le pole supérieur du rein droit en arrière et en dehors

**Figure 3:**
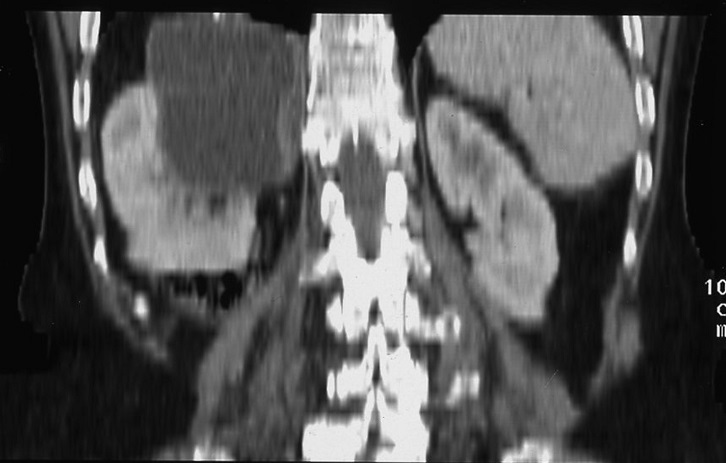
Scanner abdominal avec reconstruction coronale: une masse kystique de densité liquidienne homogène présentant une paroi propre non rehaussée après injection de produit de contraste de la loge surrénalienne droite

**Figure 4:**
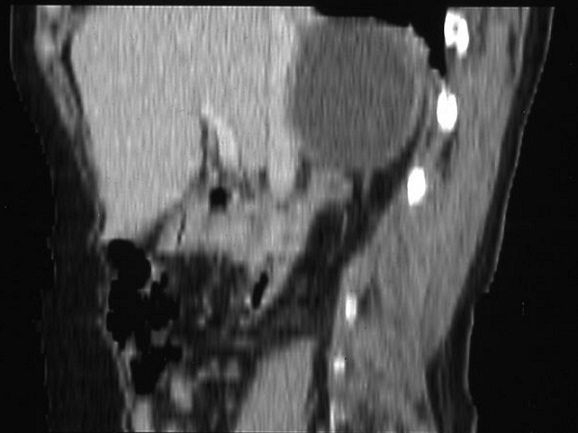
Scanner abdominal avec reconstruction sagittale: une masse kystique de densité liquidienne homogène présentant une paroi propre de la loge surrénalienne droite

Madame Z.O, âgée de 76 ans, originaire d'une zone endémique pour le kyste hydatique, opérée à deux reprises pour kystes hydatiques du foie (il y respectivement 6 ans et 3 ans), a été admise pour des douleurs de l'hypochondre droit. Son histoire remontait à 3 mois, marquée par des douleurs de l'hypochondre droit à type de pesanteur, sans autres signes accompagnateurs. L'examen clinique avait trouvé une patiente avec un état général conservé, apyrétique, anictérique, et une sensibilité à la palpation de l'hypochondre droit. Le bilan hépatique était correct et la sérologie hydatique était positive. La radiographie du thorax était normale. L’échographie abdominale avait montré une lésion kystique de l'espace hépato-rénal. Le scanner abdominal en coupe axiale sans (Figure 1) et avec (Figure 2) injection de produit de contraste avec reconstruction coronale (Figure 3) et sagittale (Figure 4) avait montré une masse kystique de densité liquidienne homogène de 50x40 mm présentant une paroi propre non rehaussée après injection de produit de contraste de la loge surrénalienne droite refoulant le pole supérieur du rein en arrière et en dehors. Le diagnostic retenu était un kyste hydatique de la surrénale droite, et la patiente a été opérée par une reprise de l'ancienne cicatrice sous-costale droite. L'exploration avait trouvé un kyste à contenu eau de roche de la loge surrénalienne droite. Après stérilisation du contenu du kyste et aspiration de la membrane proligère, il a été réalisé une résection du dôme saillant et un drainage de la cavité résiduelle par une sonde de salem. Les suites opératoires immédiates ont été favorables, le drain a été retiré à J 3 post-opératoire et la patiente a été mise sortante à J5. L’étude anatomopathologique avait conclu à un kyste hydatique de la surrénale. La patiente a été suivie à la consultation avec un recul de deux ans, les contrôles n'avaient pas révélé de récidives.

## Discussion

La localisation surrénalienne constitue une localisation extrêmement rare de la maladie hydatique représentant moins de 0.5% de l'ensemble des kystes hydatiques [1, 2]. La maladie hydatique constitue seulement 7% des kystes surrénaliens [3]. Dans une série tunisienne de 265 cas de kystes hydatiques extra-pulmonaires, BELLIL [4] a colligé 4 cas de localisation surrénalienne soit 1.5% de l'ensemble des kystes extra-pulmonaires. La plus grande série publiée dans la littérature est celle d'ACKAY en 2004, comportant neuf cas [5]. En Tunisie, HORCHANI a rapporté en 2006, une série de 6 cas sur une période de 10 ans [6]. Notre cas de kyste hydatique de la glande surrénale était le seul parmi 480 cas de kyste hydatique pris en charge dans notre service pendant une période de 16 ans, ce qui représente 0.2% des cas (Tableau 1). Toutes les tranches d’âge peuvent être touchées, mais cette localisation semble plus fréquente entre 50 et 60 ans, et légèrement plus fréquente chez le sexe féminin [5, 7], d'ailleurs, notre cas était une femme âgée de 76 ans. Mais dans la série de HORCHANI [6], on note une nette prédominance masculine (cinq hommes pour une femme). Le mécanisme de l'atteinte surrénalienne est encore mal élucidé. Plusieurs théories ont été avancées dans la littérature; la dissémination par voie artérielle semble être la théorie la plus probable [3, 8]. Ceci explique que le kyste hydatique de la glande surrénale est le plus souvent primitif. En fait, dans la plus grande série de cette localisation comportant 9 cas colligé par ACKAY [5], cinq étaient primitifs. Notre cas semble être secondaire à une localisation hépatique traitée auparavant à deux reprises. Les circonstances de découverte varient selon le stade évolutif. Le plus souvent le kyste hydatique de la surrénale est asymptomatique [3, 7–9]. La symptomatologie est dominée par les signes de compression notamment des douleurs lombaires ou de l'hypochondre droit non spécifiques, qui résument souvent le tableau clinique, ou des signes de compression gastro-intestinales. L'examen clinique peut retrouver dans certains cas une masse palpable. En cas de surinfection du kyste, il se manifeste par un tableau de suppuration profonde. L'hypertension artérielle secondaire au kyste hydatique de la surrénale, due probablement à la pression exercée par le kyste sur le parenchyme glandulaire, a été rarement décrite par TAZI [9], ESCUDERO [10] et MOKHTARI [11]. Le diagnostic précis de cette topographie est difficile malgré les moyens d'imagerie existants. Si le diagnostic de kyste hydatique est relativement facile (par l’échographie, le scanner et la sérologie hydatique), ce n'est pas le cas de sa topographie puisque les sièges rénal (kyste exophitique du pole supérieur du rein), hépatique ou même biliaire peuvent être évoqués.

**Tableau 1:** Fréquence de la localisation surrénalienne du kyste hydatique selon les auteurs

Auteurs	Nombre Total	Nombre de localisation surrénalienne	Poucentage de la localisation surrénalienne
Polat(Turquie) [1]	368	2	0.5%
Ben Ayed (Tunisie) [2]	281	1	0.35%
Notre série	480	1	0.20%

L’échographie reste le premier examen à demander pour cette localisation. La profondeur des glandes surrénales et parfois les calcifications périphériques rendent l′exploration échographique difficile. La tomodensitométrie permet alors de mieux préciser le siège et les rapports avec les organes avoisinants. Mais souvent le diagnostic de certitude n'est posé qu'en per-opératoire. Dans notre cas, la tomodensitométrie abdominale a permis de poser le diagnostic en pré-opératoire. Le diagnostic préopératoire reposant sur l'imagerie (échographie et tomodensitométrie) couplée à l'immunologie hydatique peut s'avérer difficile surtout lorsque le kyste est univésiculaire, à paroi non calcifiée avec une immunologie négative. Le diagnostic différentiel d'un kyste hydatique de la surrénale se pose avec les autres masses kystiques de la surrénale (Lymphangiome kystique, pseudo kyste hémorragique, Kyste à revêtement épithélial) et les masses kystiques extrasurrénaliennes. Le traitement de choix du kyste hydatique de la glande surrénale est chirurgical. La voie d'abord coelioscopique peut être utilisée; DEFECHEREUX [12] en 2000, DIONIGI [13] en 2007 et KUMAR en 2014 [14] ont rapporté un traitement laparoscopique de cette localisation. La laparotomie peut être par voie lombaire intercostale avec ou sans résection de la onzième cote; ou antérieure trans-péritonéale permettant un jour suffisant sur le foie en cas de localisation hépatique associée [3, 6, 7]. Dans notre cas, la voie d'abord utilisée était l'ancienne cicatrice du traitement du kyste hydatique du foie: la sous-costale droite. Il est impératif de protéger le reste de la cavité péritonéale par des champs imbibés de scolicide (eau oxygénée ou sérum hypertonique à 10 ou 20%) au pourtour du kyste hydatique, afin d'empêcher la dissémination du parasite en cas d'ouverture accidentelle du kyste lors de son exérèse. La conservation de la glande doit être la règle, sauf en cas de destruction de la surrénale par le kyste. La résection du dôme saillant du kyste avec drainage de la cavité résiduelle est le geste le plus recommandé dans la littérature [3, 5–8]. Dans notre cas, le traitement chirurgical a consisté en une résection du dôme saillant. Les suites opératoires sont le plus souvent simples. C’était le cas de notre patiente pour laquelle le suivi n'a pas détecté de récidive. La prophylaxie postopératoire par le traitement antiparasitaire (les benzimidazolés) même si elle est préconisée dans d'autres localisations par certains auteurs reste à discuter. La prévention de la contagion hydatique (par interruption du cycle parasitaire) reste une mesure indispensable pour éviter la maladie hydatique quelle que soit sa localisation.

## Conclusion

Le kyste hydatique de la surrénale reste une pathologie exceptionnelle, qu'il faudra évoquer devant toute tumeur kystique de la surrénale, particulièrement dans un pays endémique. Les aspects cliniques de cette affection sont variables et non spécifiques. La tomodensitométrie associée à la sérologie hydatique permet de poser le diagnostic. Le traitement est chirurgical et doit être au mieux conservateur. La prévention de la contagion hydatique reste une mesure indispensable pour éviter la maladie hydatique quelle que soit sa localisation.
